# Resveratrol inhibits β-amyloid-induced neuronal apoptosis via regulation of p53 acetylation in PC12 cells

**DOI:** 10.3892/mmr.2014.3034

**Published:** 2014-12-03

**Authors:** ZHIBING AI, CHENGYAN LI, LONGTI LI, GUOHOU HE

**Affiliations:** 1 Department of Neurology, Renmin Hospital of Wuhan University, Wuhan, Hubei 430060, P.R. China; 2 Department of Neurology, Taihe Hospital, Hubei University of Medicine, Shiyan, Hubei 442000, P.R. China

**Keywords:** resveratrol, apoptosis, p53, PC12 cells

## Abstract

The natural product resveratrol possesses diverse biological activities, including anti-inflammatory, anti-oxidant, anti-cancer and anti-aging effects in multiple organisms. The neuroprotective role of resveratrol has recently been reported in a cell model of amyloid (A)β(25–35)-induced neurotoxic injury using PC12 cells. However, the pathomechanism by which resveratrol inhibits neuronal apoptosis has remained to be elucidated. The present study therefore aimed to confirm the neuroprotective effects of resveratrol in an Aβ(25–35)-induced model of neurotoxicity in PC12 cells and elucidate the mechanisms underlying these effects. It was demonstrated that resveratrol exerted neuronal protection through inhibition of cell apoptosis, which was associated with an increased acetylation level of p53. In accordance with this effect, when the acetylation level of p53 was decreased by p53 acetylation inhibitor pifithrin-α, the protective effects of resveratrol were abrogated. In conclusion, it was revealed that resveratrol inhibited Aβ(25–35)-induced cell apoptosis via the acetylation of p53 in PC12 cells.

## Introduction

The natural product resveratrol was found to exhibit a diverse range of biological activities in diseases associated with oxidative stress (1). As a polyphenolic natural product, resveratrol is automatically synthesized by plants in response to fungal attack or exposure to ultraviolet light (2). In the numerous plants and organs resveratrol is produced by, it is mainly localized to the skin and seeds of purple grapes and peanuts (3). In particular, resveratrol is an active polyphenolic component present in red wine and numerous plants, which have multiple potential therapeutic benefits in the treatment of cancer, inflammation, metabolic disorders and neurological disorders. Studies have indicated that cognitive degeneration may be attenuated by regular red wine consumption, in which resveratrol contributes to the therapeutic effects (4,5).

Resveratrol is involved in anti-inflammatory, anti-oxidant, anti-cancer and anti-aging processes in multiple organisms. For example, resveratrol supplementation reduced aortic atherosclerosis and calcification and attenuated loss of aerobic capacity in a mouse model of uremia (6). In respiratory syncytial virus infection, resveratrol was reported to inhibit the Toll/interleukin-1 receptor-domain-containing adapter-inducing interferon-β-dependent pathway by upregulating sterile alpha and armadillo motif protein and thereby contributing to the anti-inflammatory effects observed (7). In adipose tissue metabolism, resveratrol increased brown adipose tissue thermogenesis markers by increasing sirtuin 1 (SIRT1) expression and energy expenditure, and decreasing fat accumulation in the adipose tissue of mice fed a standard diet (8). Recently, resveratrol has received attention in the field of neuroscience due to its neuroprotective potential (2). In stroke and Huntington’s disease, resveratrol was reported to exert neuroprotective effects (9). Resveratrol was also found to protect neurons against 1-methyl-4-phenylpyridine ion, peroxide and β amyloid (Aβ) injury (10–12). Furthermore, it was reported that in a rat model of Alzheimer’s disease (AD), resveratrol was able to prevent cognitive impairment (13). Therefore, resveratrol potentially has a pivotal role in protecting neurons against damage.

p53, a known tumor suppressor, induces cell cycle arrest and apoptotic cell death in response to DNA damage. p53 transcriptionally activates its downstream target genes, including p21 for cell-cycle arrest and B-cell lymphoma-2 protein (Bcl-2)-associated X protein (Bax) for apoptosis (14,15), whereas in mitochondria, p53-mediated apoptosis influences its own transcriptional activity as well as Bcl-2 family members (16). p53 is regulated by post-translational modifications, including phosphorylation, ubiquitination and acetylation (17), where the acetylation of p53 augments its DNA binding affinity (18). These results supported the hypothesis that modulation of the deacetylation or acetylation of p53 had a profound effect on p53 stability, as well as function. The balance of acetylation and deacetylation of p53 may be an important target in the prevention or treatment of disease.

The p53 protein has multiple acetylation sites, and its hyperacetylation is stabilized and activated endogenously to trigger apoptosis (17,19). In the present study, the acetylation level of p53 in response to resveratrol treatment was assessed. As a toxic factor, Aβ(25–35) triggers the development of multiple degenerative diseases of the nervous system and its aggregation has an important role in the initiation of the pathogenesis of such diseases (20). In the present study, the neuroprotective role of resveratrol in a toxic cell model using PC12 cells that were exposed to Aβ(25–35) injury was assessed. Subsequently, whether the neuroprotective role of resveratrol was due to the inhibition of apoptosis in PC12 cells was evaluated. Furthermore, the present study aimed to elucidate the role of p53 acetylation levels in resveratrol-mediated inhibition of apoptosis in PC12 cells.

## Materials and methods

### Cells and cell culture

The PC12 cell line was obtained from the Cell Bank at the Chinese Academy of Sciences (Shanghai, China). Cells were maintained in Dulbecco’s modified Eagle’s medium (DMEM; HyClone, GE Healthcare, Little Chalfont, UK) containing 10% fetal bovine serum (FBS; HyClone) at 37°C in a humidified atmosphere of 5% CO_2_.

### Reagents

Primary antibodies against Bax, Bcl-2 and caspase-3 were all purchased from Santa Cruz Biotechnology Inc. (Dallas, TX, USA). For the detection of transcriptional modification, primary antibodies against p53 (100 μl, No. 9282S) were purchased from Cell Signaling Technology, Inc. (Boston, MA, USA). Aβ(25–35), resveratrol, pifithrin-α and dimethyl sulfoxide (DMSO) were commercially obtained from Sigma-Aldrich (St. Louis, MO, USA). Aβ(25–35) was prepared as described previously (21). In brief, resveratrol was dissolved in DMSO at a concentration of 100 mM to produce a stock solution and stored at −20°C. The stock solution was diluted to 5 mM in serum-free DMEM prior to use and the working solution was further diluted with DMEM to the required concentrations. Aβ(25–35) was dissolved in deionized distilled water and subsequently filtered (0.22 mm filter; EMD Millipore, Billerica, MA, USA). The solution was aged by incubating at 37°C for one week and subsequently stored at −20°C.

### Experimental design

PC12 cells were cultured in 12-well plates at a density of 5×10^4^ cells/cm^2^ and then divided into four distinct groups for treatment: *i*) PC12 cells cultured in DMSO without Aβ(25–35) and resveratrol treatments (control); *ii*) PC12 cells cultured in DMSO and treated with 20 mM final concentration of Aβ(25–35) [Aβ(25–35) group]; *iii*) PC12 cells cultured in DMSO and treated with resveratrol (resveratrol group); *iv*) PC12 cells cultured in DMSO and treated with 20 mM Aβ(25–35) and resveratrol [resveratrol + Aβ(25–35) group].

The cell culture medium was refreshed every three days. The highest DMSO concentration, which had no impact on the cell viability in the culture medium, was 0.1%. Forty-eight hours after exposure to resveratrol treatment, cells were digested with trypsin and washed with cold phosphate-buffered saline (PBS; 21–040-CM; Mediatech, Inc., Manassas, VA, USA) three times for subsequent analysis.

### Cell viability assay

Cell viability was assessed via colorimetric assay using the Cell Counting kit-8 (CCK-8 kit; MAB5963; Abnova, Taipei, Taiwan). Briefly, PC12 cells were washed with PBS and suspended at a final concentration of 4×10^4^ cells/ml in an assay medium and dispensed into 96-well plates. CCK-8 solution was added to cells in each well to a final concentration of 0.5 mg/ml and incubated at 37°C for 5 h. Then the medium was gently aspirated and DMSO was added to each well in order to dissolve the formazan product at room temperature. The absorbance of each sample at a wavelength of 490 nm (A490) was detected using a synergy 2 multi-mode microplate reader (Bio-Tek Instruments, Winooski, VT, USA). Experiments were performed in triplicate and cell viability was quantified based on the A490 value.

### Western blot analysis

PC12 cells were harvested and lysed using radioimmunoprecipitation assay lysis buffer (Beyotime Institute of Biotechnology, Nantong, China). Total proteins were extracted and quantified using a bicinchoninic acid kit (Boster Biological Technology, Wuhan, China). Subsequently, 50 μg proteins were fractionated using 10% SDS-PAGE (GE Healthcare, Logan, UT, USA) and electro-transferred onto nitrocellulose (NC) membranes (Bioleaf Biotech, Shnghai, China; in an ice-water bath. NC membranes were blocked with 5% skimmed milk in Tris buffer (Sigma-Aldrich) containing 0.1% Tween-20 and then incubated with the following rabbit monoclonal primary antibodies at 4°C overnight: Anti-Bax (sc-526), anti-Bcl-2 (sc-492), anti-extracellular-signal-regulated kinase (ERK; sc-292838), anti-phosphorylated (p)-ERK (sc-13073), anti-caspase-3 (all Santa Cruz Biotechnology Inc.), and anti-p53 (9282S), anti-Akt (9272) and anti-p-Akt (9275) (Cell Signaling Technology, Inc.) (all 1:1,000 dilution). Subsequently, the blots were washed and incubated with goat anti-rabbit horseradish peroxidase-conjugated immunoglobulin G secondary antibody (Santa Cruz Biotechnology) at room temperature for 1 h. Finally, blots were visualized with enhanced chemiluminescence reagent (EMD Millipore).

### Cell apoptosis analysis

PC12 cells in each group were stained with propidium iodide at a concentration of 10 mg/ml for 20 min. Cells were then labeled and observed under an LSM 780 Laser Scanning Confocal Microscope (Carl Zeiss AG, Oberkochen, Germany). In order to further distinguish early- from late-stage apoptosis and perform a quantitative analysis, flow cytometry with Annexin V-fluorescein isothiocyanate (FITC) staining was employed as previously described (19). Briefly, PC12 cells were diffused with 0.05% trypsin (Pierce Biotechnology Inc., Rockford, IL, USA), centrifuged at 189 × g for 5 min and then washed twice with sterile PBS. Subsequently, binding buffer (Fermentas, Thermo Fisher Scientific, Waltham, MA, USA) was added to cells and 6×10^5^ cells were re-suspended in the buffer, following which they were stained with Annexin V-FITC (Bioteool, Houston, TX, USA) for 15 min in the dark at room temperature. Finally, the fluorescence of each group was determined by flow cytometry (653158, BD Biosciences, Franklin Lakes, NJ, USA).

### Statistical analysis

Values are expressed as the mean ± standard error of the mean of three independent experiments. Student’s t-test was used for quantitative data analysis. SPSS version 16.0 (SPSS, Inc., Chicago, IL, USA) was used for all statistical analysis. P<0.05 was considered to indicate a statistically significant difference between values.

## Results

### Resveratrol prevents apoptotic cell death induced by Aβ(25–35) in PC12 cells

**Figure 1:**
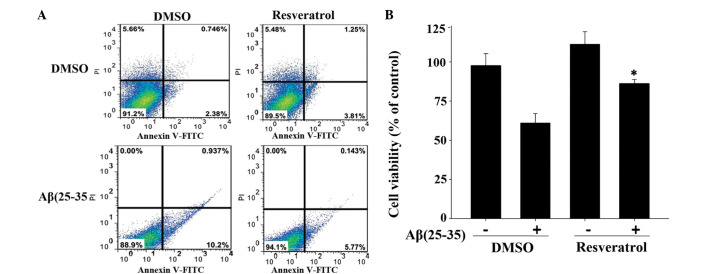
Resveratrol prevents Aβ(25–35)-induced apoptotic cell death in PC12 cells. (A) Flow cytometry was used to examine the effects of resveratrol on Aβ(25–35)-induced cell apoptosis. In each image, the lower left quadrant area indicates the survival of cells, the lower right quadrant indicates the level of early-stage apoptosis and the upper right quadrant indicates the level of late-stage apoptosis. (B) A CCK-8 assay was used to evaluate cell viability. Resveratrol inhibited Aβ(25–35)-induced cell apoptosis. Values are presented as the mean ± standard error of the mean of three independent experiments. ^*^P<0.05 between the Aβ(25–35) injury group and the revesterol + Aβ(25–35) group. DMSO, dimethyl sulfoxide; FITC, fluorescein isothiocyanate; Aβ, β amyloid.

The Aβ(25–35) peptide is a hallmark of degenerative disorders, in particular AD (22). As a toxic factor, abnormal deposits of Aβ(25–35) protein in the brain have a critical role in the pathogenesis of multiple diseases. To examine the role of resveratrol in preventing neurons from undergoing cell death, a cell model of Aβ(25–35) injury was constructed in PC12 cells. Cells were divided into four groups with each group treated with DMSO, Aβ(25–35), resveratrol or Aβ(25–35) in combination with resveratrol, respectively. The protective effects of resveratrol against cell apoptosis of PC12 cells were evaluated using flow cytometry with Annexin V-FITC staining, which also allows efficient determination of early- and late-stage apoptosis. When exposed to 20 mM Aβ(25–35), apoptosis was induced in PC12 cells compared with the apoptotic rate of the normal control group (P<0.05). Furthermore, Aβ(25–35) induced early- as well as late-stage apoptosis as compared with apoptotic rates of the control group [Fig. 1A; early stage, 10.2% in Aβ(25–35) group vs. 2.38% in control group; late stage, 0.937% in Aβ(25–35) group vs. 0.746% in control group]. Of note, when resveratrol was added to PC12 cells, a significant reduction in cell apoptosis was observed. Furthermore, early- and late-stage apoptosis were markedly inhibited [Fig. 1A; early stage, 5.77% in resveratrol + Aβ(25–35) group vs. 10.2% in Aβ(25–35) group; late stage, 0.143% in resveratrol + Aβ(25–35) group vs. 0.937% in Aβ(25–35) group].

**Figure 2:**
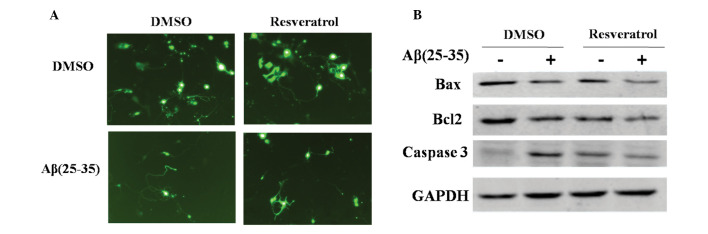
Resveratrol inhibits apoptotic inducers and promotes apoptotic inhibitors in PC12 cells. (A) Cell growth and morphology of each group. Cells receiving Aβ(25–35) treatment presented retracted neurites and decreased cell confluence due to loss of cells (lower left and right panels). Resveratrol-treated cells presented recovered neutites and cell growth confluence (right upper and right lower panels). (B) Resveratrol treatment inhibited the apoptotic inducers, Bax and caspase-3, and the apoptotic inhibitor, Bcl-2, in PC12 cells. Aβ, β amyloid; DMSO, dimethyl sulfoxide; Bcl-2, B-cell lymphoma-2 protein; Bax, Bcl-2-associated protein.

A CCK-8 assay was subsequently employed to evaluate cell viability. The addition of Aβ(25–35) to PC12 cells significantly decreased cell viability from nearly 100% to ~60% [Fig. 1B; DMSO panels, Aβ(25–35)(+) vs. Aβ(25–35)(−)]. However, when PC12 cells were co-treated with resveratrol and Aβ(25–35), cell viability was significantly increased from 60% in the Aβ(25–35) injury group to nearly 90% in the resveratrol + Aβ(25–35) group (Fig. 1B; P<0.05). In addition, marked survival of PC12 cells exposed to Aβ(25–35) was observed following treatment with resveratrol (Fig. 2A). As indicated in Fig. 2A, PC12 cells developed long neurites following culture in DMSO (Fig. 2A, left upper panel); when exposed to Aβ(25–35), the neurites of cells retracted gradually and cell death was apparent due to markedly decreased cell confluence. The neurites gradually disappeared, while cell debris appeared (Fig. 2A, left lower panel). Following treatment with resveratrol, a protective effect on PC12 cells was observed, identified by the rescued cell growth and morphology (Fig. 2A, right panel). These results confirmed that resveratrol was able to inhibit Aβ(25–35)-induced apoptotic cell death. In conclusion, resveratrol prevented Aβ(25–35)-induced cell apoptosis in PC12 cells.

### Resveratrol inhibits apoptotic inducers and promotes apoptotic inhibitors

To further confirm that resveratrol was able to prevent PC12 cells from apoptosis induced by Aβ(25–35), western blot analysis was used to evaluate expression of apoptotic inducers and inhibitors. Aβ(25–35) induced activation of the apoptotic inducer, caspase-3 [Fig. 2B; Aβ(25–35)(+) lane vs. Aβ(25–35)(−) lane in DMSO group]. Concomitantly, resveratrol decreased Bax and caspase-3 expression, as well that of the apoptotic inhibitor, Bcl-2. These results further confirmed that resveratrol inhibited Aβ(25–35)-induced cell apoptosis in PC12 cells.

### Inhibition of apoptosis by resveratrol is associated with increased acetylation level of p53

**Figure 3:**
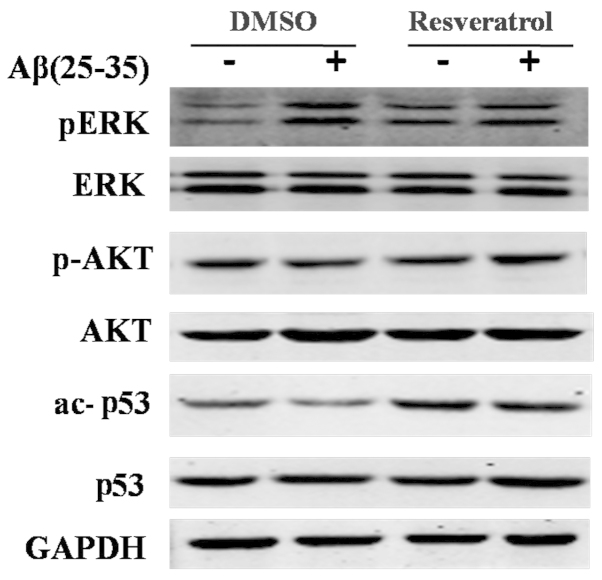
Inhibition of apoptosis by resveratrol is associated with an increase in p53 acetylation levels. Expression levels of ERK, Akt and p53, as well as their common translational modifications were detected. ac-p53 levels were recovered and increased markedly following resveratrol treatment by comparing ac-p53 levels in the resveratrol + Aβ(25–35) group with those in the Aβ(25–35) group. DMSO, dimethyl sulfoxide; pERK, phosphorylated extracellular-signal-regulated kinases; ac-P53, acetylated p53; Aβ, β amyloid.

To assess the mechanism underlying the protective effect of resveratrol in PC12 cells, the expression of proteins in several common pathways, including ERK, Akt and p53, were analyzed. p-ERK and p-Akt, as well as acetylated p53 (ac-p53) were also assessed. Akt has an upstream function of p53. The activation of Akt depends on its phosphorylation state, and phosphorylated Akt is able to interrupt the stability and activity of p53 (23). As depicted in Fig. 3, no difference in the total protein levels of ERK and p53 was detected amongst the groups. Similarly, no significant difference was detected in p-Akt expression. Expression levels of pERK were markedly increased in the Aβ(25–35) injury group. However, no significant alteration in pERK expression levels was detected in response to resveratrol treatment, which indicated that the protective effects of resveratrol against Aβ(25–35) likely had no association with ERK and Akt or their phosphorylated modifications. In the DMSO-treated cells, Aβ(25–35) significantly decreased ac-p53 expression levels, which suggested that Aβ(25–35)-induced apoptosis was associated with decreased acetylation of p53. Of note, it was demonstrated that ac-p53 levels recovered and markedly increased following resveratrol treatment (Fig. 3). Therefore, resveratrol potentially inhibited Aβ(25–35)-induced apoptosis by association with increased acetylation levels of p53.

### Inhibition of p53 acetylation abrogates resveratrol-mediated apoptosis inhibition

**Figure 4:**
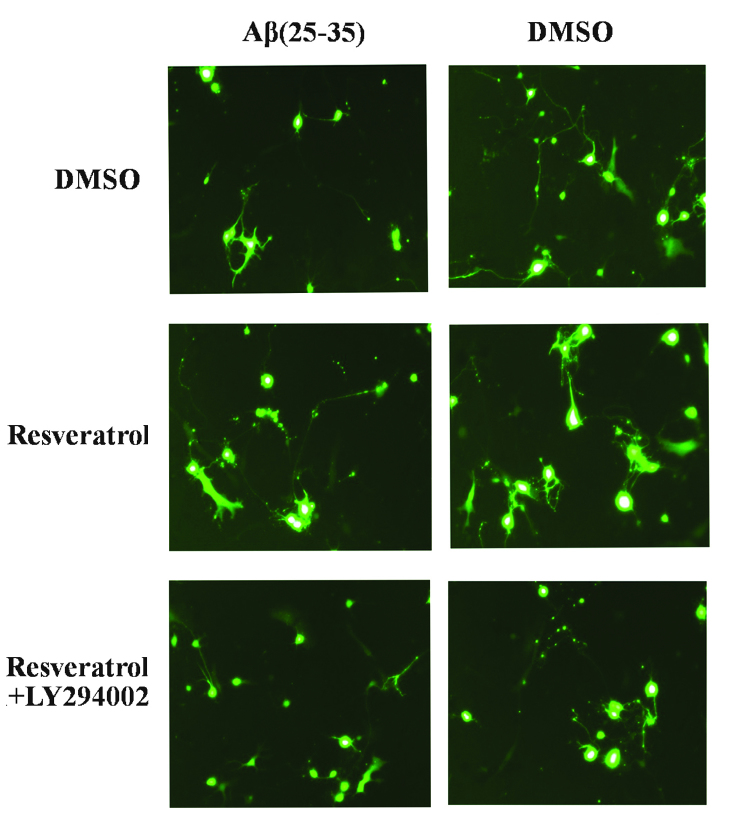
Inhibition of p53 acetylation abrogates resveratrol-mediated apoptosis inhibition. Pifithrin-α treatment was used to inhibit acetylation of p53 and resulted in attenuation of resveratrol-inhibited apoptosis. Resveratrol treatment promoted cell growth with natural neurites and adequate cell confluence (middle images). However, pifithrin-α treatment resulted in cell death with retracted neurites and damaged cell growth confluence (bottom images).

To further confirm the hypothesis that resveratrol-mediated inhibition of apoptosis in PC12 cells was positively associated with increased acetylation levels of p53, pifithrin-α, an inhibitor of p53 acetylation, was applied to the cells. Pifithrin-α is a chemical inhibitor of p53 that has been shown to protect mice from the side effects of cancer therapy (24). PC12 cells were treated with pifithrin-α to inhibit p53 expression, and subsequent alterations in cell survival were evaluated. Aβ(25–35) treatment induced cell death and caused retracted cell neurites, whereas resveratrol abrogated this Aβ(25–35)-induced apoptotic effect (Fig. 4; top and middle images). When PC12 cells were co-treated with pifithrin-α and resveratrol, cell growth was perturbed and neurites were no longer present. Cell growth and confluence were markedly decreased as indicated by a loss of cells. These results suggested that pifithrin-α attenuated the protective effects of resveratrol in PC12 cells, indicating that decreased acetylation levels of p53 may attenuate resveratrol-mediated inhibition of cell apoptosis. It may therefore be concluded that resveratrol-mediated inhibition of Aβ(25–35)-induced apoptosis was associated with an increased acetylation level of p53.

## Discussion

In the present study, a neurotoxic cell model in PC12 cells was established by administration of Aβ(25–35), which provided novel evidence for the protective effects of resveratrol against Aβ(25–35)-induced neurotoxicity. Resveratrol protected PC12 cells from neuronal damage through inhibition of apoptotic cell death. Resveratrol reversed the Aβ(25–35)-induced decreased cell viability and cell apoptosis. In particular, the underlying mechanism that contributed to resveratrol-mediated inhibition of apoptosis was examined. It was demonstrated that the neuroprotective effects of resveratrol were associated with increased acetylation levels of p53. Therefore, the neuroprotective effects of resveratrol against Aβ(25–35) in PC12 cells may be partially mediated by the acetylation of p53.

Resveratrol is a phytoestrogen, originally derived from plants, with diverse anti-proliferative and pro-apoptotic effects (25). The mechanisms underlying these effects comprise downregulation of apoptosis inhibitors, including survivin2 and Bcl-2, as well as upregulation of apoptosis inducers, including Bax (25). In the central nervous system, it was recently demonstrated that resveratrol decreased Bcl-2 expression and viability in GH3 pituitary adenoma cells of rats (26). In the present study, it was also confirmed that the protective effect of resveratrol in preventing neuronal apoptosis was associated with pro-apoptotic/anti-apoptotic factors. When the neurotoxic factor Aβ(25–35) was added to PC12 cells, cell viability was significantly decreased by ~40%. However, when PC12 cells were co-treated with resveratrol, cell viability was significantly increased from 60% in the Aβ(25–35) group to nearly 90% in that of the resveratrol + Aβ(25–35) group. In addition, a marked increase in the survival of PC12 cells was evident following resveratrol treatment. Resveratrol treatment rescued PC12-cell survival, attenuating the neuronal damage induced by Aβ(25–35). The evaluation of apoptosis-associated protein expression revealed that resveratrol treatment inhibited the expression of the pro-apoptotic protein, caspase-3, as well as that of the anti-apoptotic protein, Bcl-2. These results confirmed that resveratrol inhibited Aβ(25–35)-induced apoptotic cell death, and that apoptosis-associated proteins Bax, Bcl-2 and caspase-3 were involved in mediating the resveratrol-induced inhibition of apoptosis in PC12 cells.

Furthermore, it was demonstrated that the inhibition of apoptosis by resveratrol was associated with an increased acetylation level of p53. While total p53 remained stable in PC12 cells regardless of which treatment was administered, the acetylation level of p53 varied between groups. Aβ(25–35) treatment decreased the acetylation level of p53, which represented a process underlying Aβ(25–35)-induced apoptosis. Of note, when PC12 cells were co-treated with resveratrol, the acetylation level of p53 markedly increased, indicating that resveratrol may inhibit Aβ(25–35)-induced apoptosis via an association with the modification of p53 acetylation. In order to further analyze the involvement of p53 in mediating the effects of resveratrol, p53 inhibitor pifithrin-α was introduced. Pifithrin-α prevented the resveratrol-mediated recovery of Aβ(25–35)-induced cell growth inhibition. This result supported the conclusion that resveratrol inhibited Aβ(25–35)-induced apoptosis, potentially via the regulation of acetylation of p53.

Previous studies revealed that resveratrol upregulated SIRT1 expression in Aβ(25–35)-treated cells (27,28). SIRT1 is a nicotinamide adenine dinucleotide-dependent histone deacetylase, which has a critical role in regulating cellular activities, including transcriptional silencing of telomeres and life-span extension (29,30). Furthermore, p53 was found to be regulated by SIRT1 (31,32), hence it may be possible that resveratrol is able to cross-talk with SIRT1 and p53. Resveratrol may influence the acetylation level of p53 via the regulation of SIRT1 expression. Further study is required in order to investigate this possible interaction.

In conclusion, the results of the present study provided novel evidence which indicated that resveratrol, a natural product, exerted a protective effect on PC12 cells in an Aβ(25–35)-induced cell model of neurotoxic damage. Resveratrol inhibited Aβ(25–35)-induced cell apoptosis and therefore promoted cell viability. The inhibition of Aβ(25–35)-induced apoptosis by resveratrol may be associated with an increased acetylation level of p53. These results may provide a basis for elucidating the therapeutic potential of resveratrol in treating degenerative disorders of the brain.
